# *De Novo* Donor Specific Antibody and Long-Term Outcome After Liver Transplantation: A Systematic Review and Meta-Analysis

**DOI:** 10.3389/fimmu.2020.613128

**Published:** 2020-12-23

**Authors:** Zahra Beyzaei, Bita Geramizadeh, Zahra Bagheri, Sara Karimzadeh, Alireza Shojazadeh

**Affiliations:** ^1^ Transplant Research Center, Shiraz University of Medical Sciences, Shiraz, Iran; ^2^ Department of Pathology, Medical School of Shiraz University, Shiraz University of Medical Sciences, Shiraz, Iran; ^3^ Department of Biostatistics, Shiraz University of Medical Sciences, Shiraz, Iran; ^4^ Shiraz Medical School Library, Shiraz University of Medical Sciences, Shiraz, Iran; ^5^ Student Research Committee, Shiraz University of Medical Sciences, Shiraz, Iran

**Keywords:** *de novo* donor-specific anti-human leukocyte antigen antibodies, liver transplantation, humoral rejection, acute antibody-mediated rejection, human leukocyte antigen single antigen bead

## Abstract

**Background:**

The impact of *de novo* anti-HLA donor-specific alloantibodies (DSA) which develop after long-term liver transplantation (LT) remains controversial and unclear. The aim of this study was to investigate the role of *de novo* DSAs on the outcome in LT.

**Methods:**

We did a systematic review and meta-analysis of observational studies published until Dec 31, 2019, that reported *de novo* DSA outcome data (≥1 year of follow-up) after liver transplant. A literature search in the MEDLINE/PubMed, EMBASE, Cochrane Library, Scopus and Web of Science Core Collection databases was performed.

**Results:**

Of 5,325 studies identified, 15 fulfilled our inclusion criteria. The studies which reported 2016 liver transplant recipients with *de novo* DSAs showed an increased complication risk, i.e. graft loss and chronic rejection (OR 3.61; 95% CI 1.94–6.71, *P* < 0.001; I^2^ 58.19%), and allograft rejection alone (OR 6.43; 95% CI: 3.17–13.04; *P* < 0.001; I^2^ 49.77%); they were compared to patients without *de novo* DSAs. The association between *de novo* DSAs and overall outcome failure was consistent across all subgroups and sensitivity analysis.

**Conclusions:**

Our study suggested that *de novo* DSAs had a significant deleterious impact on the liver transplant risk of rejection. The routine detection of *de novo* DSAs may be beneficial as noninvasive biomarker-guided risk stratification.

## Introduction

The damaging effect of alloantibodies against donor HLA has been widely known in all solid organ transplantation, except the liver (1, 2). It is clearly documented that developing *de novo* donor specific antibodies (dn-DSA) has a pathogenic role in solid organ allografts such as the kidney (3), lung (4), heart (5), pancreas (6), and intestine (7). dn-DSAs are associated with late acute antibody mediated rejection and chronic antibody-mediated rejection, which can have effects on the long survival (8–10).

Since the early 1990s, the liver has been known as an immunologically resistant organ to rejection in transplantation (11). The cause of this resistance was considered because of the great ability of the liver to absorb or neutralize alloantibodies directed against HLA antigens (12). In liver transplant (LT) recipients, the first reports failed to reveal an association between dn-DSAs and graft rejection or survival (13–15). Nowadays, improved understanding of the humoral response in solid organ transplants has been greatly increased by new sensitive, high-throughput, and cutting-edge facilities in antibody detection (13, 16–19). Therefore, further reports demonstrate that LT recipients who develop dn-DSA reveal lower graft and patient survival (20, 21). Furthermore, the latest studies suggested the possible impact of dn-DSA and the humoral response as a risk factor for unexplained post-operative complications such as biliary problems, acute and chronic rejection, and patient survival, especially in deceased-donor liver transplantation (DDLT) compared to living donor liver transplantation (LDLT) (22–27). In LT, the above outcomes are often observed in biopsies from pediatric or adult patients, and the severity of worse outcome correlates with the timing from LT to applying the biopsy procedure and follow up (28). Nevertheless, uncertainty about the role of dn-DSAs after liver transplantation in humoral response still exists, and dn-DSA monitoring has not been universally adopted by all transplant centers in LT recipients (27, 29).

Consequently, the main question of our study is “Is dn-DSA important and effective in long term liver injury in LT patients?” To date, few studies have assessed the incidence and effect of dn-DSA on liver-transplant patients. Because of the lack of consensus on this topic, the aim of this systematic review and meta-analysis was to evaluate the role of dn-DSAs on the graft outcome in long-term LT recipients. The primary outcome measures the overall graft loss and chronic rejection results. Secondary outcomes included the estimated graft loss and chronic rejection separately. Subgroup and sensitivity analysis of the main outcomes were stratified.

## Materials and Methods

This systematic review and meta-analysis was carried out according to the recommendations by the Cochrane Collaboration, the PRISMA statement and Meta-analysis of Observational Studies in Epidemiology guidelines (30–32). This study is registered with PROSPERO, number CRD42020172054.

### Data Sources and Search Strategy

Two reviewers (ZBe and SK) conducted a systematic literature search independently in the MEDLINE/PubMed, EMBASE, Cochrane Library, Scopus and Web of Science Core Collection databases with no time and language restrictions up to Dec 31, 2019. Bibliography of the selected articles on the topic and other relevant systematic reviews were manually searched for additional studies and for minimizing the publication bias. The search strategy was designed using controlled keywords and the MeSH terms (Medical Subject Heading). One word (keywords) was identified by examining relevant references in the literature and the Medical Subject Headings (MeSH) used by a specialist librarian (SK) who has extensive experience in systematic reviews from EMBASE and MEDLINE (http://www.nlm.nih.gov/mesh/). The following main key search terms were used for database search: “liver transplantation”, “donor specific anti-HLA antibodies”, “anti-HLA DSAs”, “human leukocyte antigen”, “solid phase assay”, “outcome”, “graft loss”, “graft survival”, and “rejection”. To do it comprehensively, we also reviewed all references of full-text articles to find relevant studies, which might have been missed in our search strategy. See **Table S1** for the full search strategy.

### Study Selection and Criteria

At first, studies of any relevant design and in any language on the impact of dn-DSA and/or complement-activating anti-HLA DSAs on long-term graft outcome in both adult and pediatric patients were selected. The inclusion criteria were as follows: (1) All articles designed to assess the association of dn-DSA with allograft outcomes (protocol DSA screening); (2) The patients being screened for dn-DSA at least one time on the day of transplantation (Day 0) and another screening after LT; (3) Studies reporting positive and negative dn-DSA liver transplant with/or without outcome after LT; (4) If a non dn-DSA control group was included, it reported dn-DSA with/or without outcome after LT; (5) The outcome should be graft loss and/or graft rejection in LT; (6) The long-term follow-up post-transplantation in studies was considered, which should be more than 1 year; (7) All articles just reporting dn-DSA detection by the Luminex single-antigen bead (SAB) technique (33, 34); and (8) Graft rejection was confirmed by liver biopsy (35).

Exclusion criteria were: (1) Case reports, editorials, reviews, and letters, animal studies, conference papers, non-liver solid organ transplant studies; (2) Combined transplanted patients, re-transplanted patients or second liver transplant cases; (3) Articles not exactly determining which of these cases was dn-DSA or performed DSA; and (4) Articles just reporting T-cell-mediated rejection. If multiple papers reported on similar patients from one center, the most complete publication was chosen for advanced synthesis and another one was excluded unless they were different in data.

The corresponding author of each suspected study was asked to verify dn-DSA detection when these were not available on the manuscript. We sent two separate reminders unless we obtained a certain answer. When no reply was received, the study was excluded from the analysis.

Two authors (ZBe and BG) independently assessed the potential eligibility of the articles in two stages: screening of titles and abstracts and screening of full-text articles using the eligibility criteria, as described above. The selected studies were fully read by both reviewers and those which fulfilled the eligibility criteria were selected for detailed data mining and quality assessment. Inter-rater reliability (kappa statistic) was calculated using the MedCalc software 19.1. Disagreements at both stages were resolved by consensus and referred back to the original article.

### Data Extraction and Quality Assessment

Two reviewers (ZBe and BG) extracted the data; ZBe extracted data from the articles and BG rechecked them for accuracy. The collected data included the first author’s name; publication year; sample size; design of the study; period of inclusion; median time of the last biopsy to analysis dn-DSA; mean patient follow-up time post-LT; type of organ donation; ABO blood type; potential confounding factors; population and center characteristics; etiology of liver disease; recipient Model for End-Stage Liver Disease (MELD) or Child-Pugh score at transplant; positive dn-DSA patients with outcome (rejection, graft loss); positive dn-DSA patients without outcome (rejection, graft loss); negative dn-DSA patients with outcome (rejection, graft loss); negative dn-DSA patients without outcome (rejection, graft loss); MFI value; and the outcome of graft loss or/and graft rejection. The raw data were extracted using binary analysis.

For quality assessment, the Newcastle-Ottawa scale assessed the quality of the studies for both cohort and case-control studies. The quality can be given eight stars in the Selection (up to 4 stars), Comparability (1 star) and Outcome (up to 3 stars) categories. Articles could receive one additional star from comparability if they analyzed not only dn-DSA, but also complement-activating donor-specific anti-HLA antibodies. Therefore, the maximum star could be nine stars. The two authors (ZBe, BG) independently scored the publications, and disagreement was resolved by discussion and consensus. Meanwhile, as all the included articles were observational studies, the context and population were also considered. See **Table S2** regarding the NOS scoring system.

### Data Synthesis and Statistical Analysis

In the present study, meta-analysis was performed based on random effect models in order to control the between-study heterogeneity and within-study heterogeneity. In the first step, we considered all eligible studies in the analysis for rejection outcome and graft loss. The rate of rejection and/or graft loss was compared between positive dn-DSA patients with LT and negative dn-DSA ones based on odds ratio. Publication bias was visually assessed using a funnel plot. In addition, heterogeneity was tested using Q Cochran heterogeneity test and I^2^ index was reported for measuring the degree of heterogeneity. This index provides information regarding the percentage of total variation across different studies due to heterogeneity rather than chance. A value of 0% indicates no observed heterogeneity; values greater than 50% indicate high heterogeneity and consequently considerable caution should be considered in interpreting the results. We reported odds ratio with 95% confidence interval as a binary outcome data in all cases. The statistical analysis was performed by RevMan software, version 5.1 and MedCalc software 19.1.

## Subgroup and Sensitivity Analyses

These analyses were conducted in different subgroups in order to consider different sources of heterogeneity regarding the primary outcome. The following subgroup analyses were considered: adult vs. pediatric, living vs. deceased donors, duration of follow-up post-transplantation, type of biopsy, potential confounding factors, cutoff MFI comparison, and highly methodological quality studies. The center effect was also studied based on the exclusion of the larger studies.

## Results

### Study Identification and Characteristics

**Figure 1:**
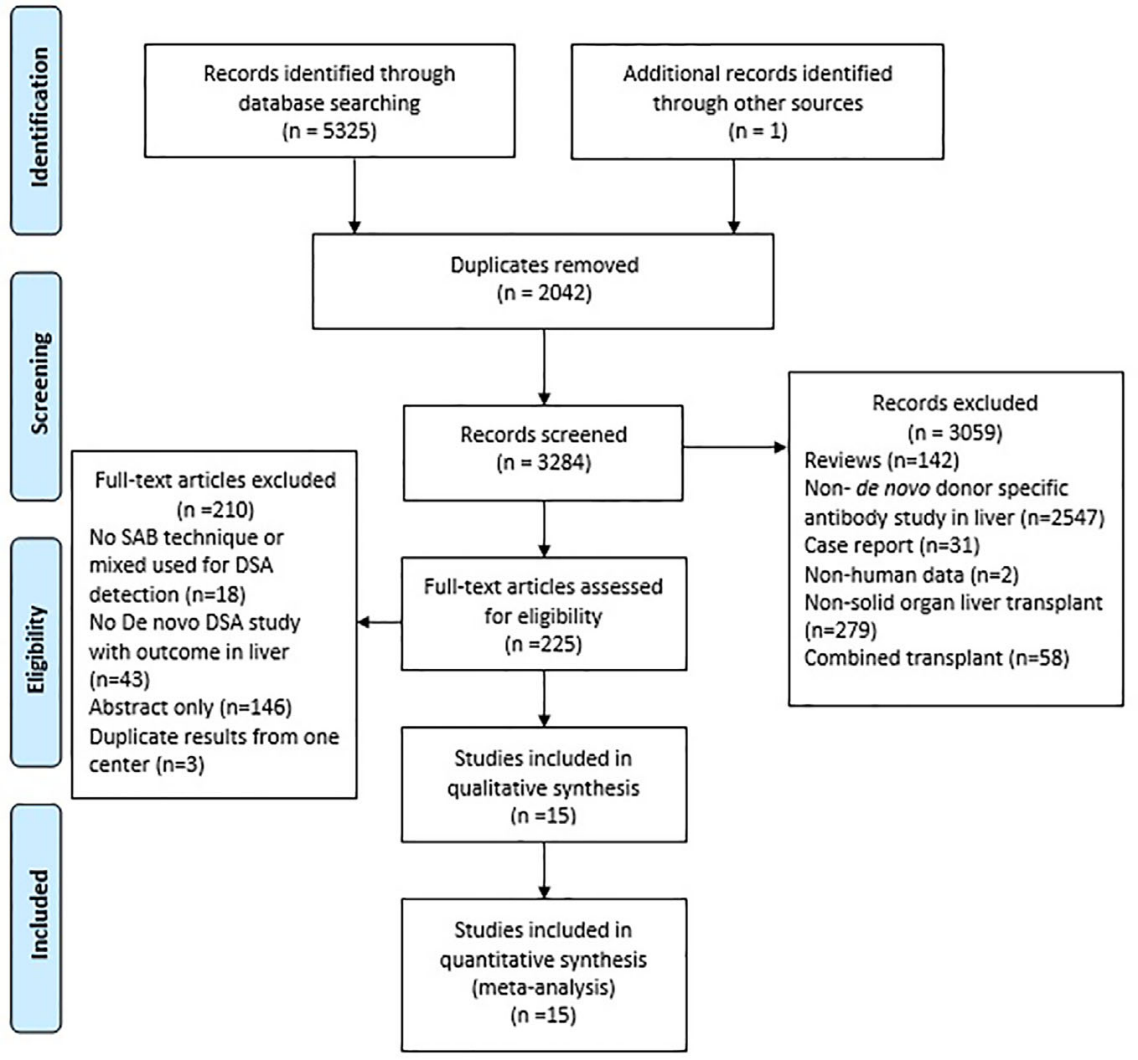
The flow diagram of the study selection for the systematic review.

The electronic search identified 5,325 potentially relevant citations. The 225 full-text articles were found for possible eligibility with regard to the inclusion criteria. Finally, 15 studies and 2016 patients were included in the final systematic review and meta-analysis, including 5 studies with data on the allograft loss (36–40), and 10 studies with data on rejection (41–50). A flow diagram of the article selection process is demonstrated in **Figure 1**. All studies had a Newcastle-Ottawa Scale score of 6 or more, as shown in **Table 1** and detailed **Table S2**. The Kappa statistics for the study eligibility was 0.93 between the two reviewers (SE = 0.09). **Table 1** summarizes the characteristics of the included studies.

**Table 1:** General characteristics of the included studies.

First author (year)	Country	Type of study	No. Patients	Mean age (years)	Period of inclusion	Mean follow up duration (years)	Mean periods from LT to last biopsy, (month)	Population characteristics	Outcome	Donor type	Newcastle-Ottawa score
Papachristou et al. (36)	Greece	cohort	65	52.3	2010–2017	1.5	6	Retrospective; single-center study; adult patients	graft loss	DD	7
Jucaud et al. (41)	USA	cohort	40	54.5	2005–2015	5.0	12	Prospective; multicenter; randomized ITN030ST trial; achieved stable IS monotherapy (40 stable patients) were randomized to IS maintenance (n=9) or IS minimization (n=31)	rejection	NR	6
Vandevoorde et al. (37)	France	cross-sectional	292	52.4	2000–2010	7.4	6	Retrospective; single-center analysis; adult patients	graft loss	DD	9
Tokodai et al. (43)	Japan	cohort	18	2.9	1991–2013	13.0	124	Retrospective; single-center analysis; pediatric patients	rejection	LD	7
Kubal et al. (38)	USA	cohort	80	55.0	2010–2011	6.8	5	Prospective; single-center analysis; adult patients	graft loss	NR	8
Kovandova et al. (42)	Czech	cross-sectional	123	56.7	2015–2017	1.0	8	Retrospective; single-center analysis; adult patients	rejection	DD	9
den Dulk et al. (40)	Netherland	case-control	68	55.1	2000–2014	1.0	5.5	Retrospective; two-center analysis; all adult NAS patients was confirmed with direct cholangiography	graft loss	DD	8
San Segundo et al. (44)	Spain	cross-sectional	28	55.1	2002–2014	6.4	0	Retrospective; single-center analysis; adult patients	rejection	NR	6
Levitsky et al. (45)	USA	cohort	195	51.0	2004–2010	6.0	6	Retrospective; multicenter; adult patients	rejection	129 LD,66 DD	8
Ueno et al. (46)	Japan	cohort	23	2.6	1998–2009	9.7	60	Retrospective; single-center analysis; pediatric patients	rejection	LD	8
Grabhorn et al. (47)	Germany	cohort	43	10.3	1992–2012	5.0	88	Retrospective; pediatric recipients; single center study, 40% from other country	rejection	16 LD, 27 DD	9
Del Bello et al. (48)	France	cohort	152	52.1	2008–2013	2.83	12	Retrospective; single-center study; adult patients	rejection	NR	9
Kaneku et al. (39)	USA	cohort	749	52.4	2000–2009	5.0	10	Retrospective; single center study; adult liver transplant recipients	graft loss	NR	9
Miyagawa-Hayashino et al. (49)	Japan	cohort	67	2.3	1990–2011	11.1	132	Retrospective; pediatric recipients; single center study; with showing fibrosis	rejection	LD	8
O’Leary et al. (50)	USA	cohort	73	43.0	1985–NR	5.0	12	Prospective collection; single-center analysis; adult patients and 1-year survival post liver transplantation	rejection	NR	8

DD, Deceased donor; LD, Living donor; NR, not reported.

Overall, 7 (46.7%) studies originated from Europe, 5 (33.3%), from USA, and 3 (20.0%) from Japan. All studies had collected patients between Jan 20, 1985, and Dec 1, 2017 and none of them included patients with combined transplantation. Of these, 4 studies had reported pediatric liver transplant and 11 studies were on adult recipients. The number of pediatric and adult recipients of liver transplant were 151 and 1,865 for the included studies. The mean age of the pediatric and adult participants was 4.52 and 48.23 years, respectively. Seven studies had reported the transplant type (living donor vs. deceased donor) which were received from 237 living donors, and 546 deceased donors; 1,233 of them had not reported the donor type. The mean patient follow-up period post-transplantation was 5.71 years. In addition, the mean period from LT to DSA evaluation and latest liver biopsy was 33 months. Complement activating alongside the dn-DSA was also reported by their capacity to bind to C1q (4 studies), C3d (4 studies), and by their IgG subclass composition (2 studies). Model for End-Stage Liver Disease (MELD) score had been reported in 5 studies with a median of 19.

None of the studies included was sponsored or conducted by diagnostic companies involved in detection of antibodies by solid phase assay. Nine corresponding authors of the articles were contacted and asked for more **Supplementary Data**, and 5 of them provided the requested information.

### Outcomes

Overall, DSA was analyzed in 2016 liver transplants. A total number of 358 patients were found to have dn-DSA, 142 of whom were positive dn-DSA with overall outcome (rejection, graft loss); 216 patients were positive dn-DSA without outcome. A total number of 1,658 were found to have no dn-DSA, 247 of whom were negative dn-DSA patients with outcome; 1,411 patients were negative dn-DSA patients without outcome.

#### *De Novo* Donor Specific Anti-HLA DSA Status and Overall Outcome

**Figure 2:**
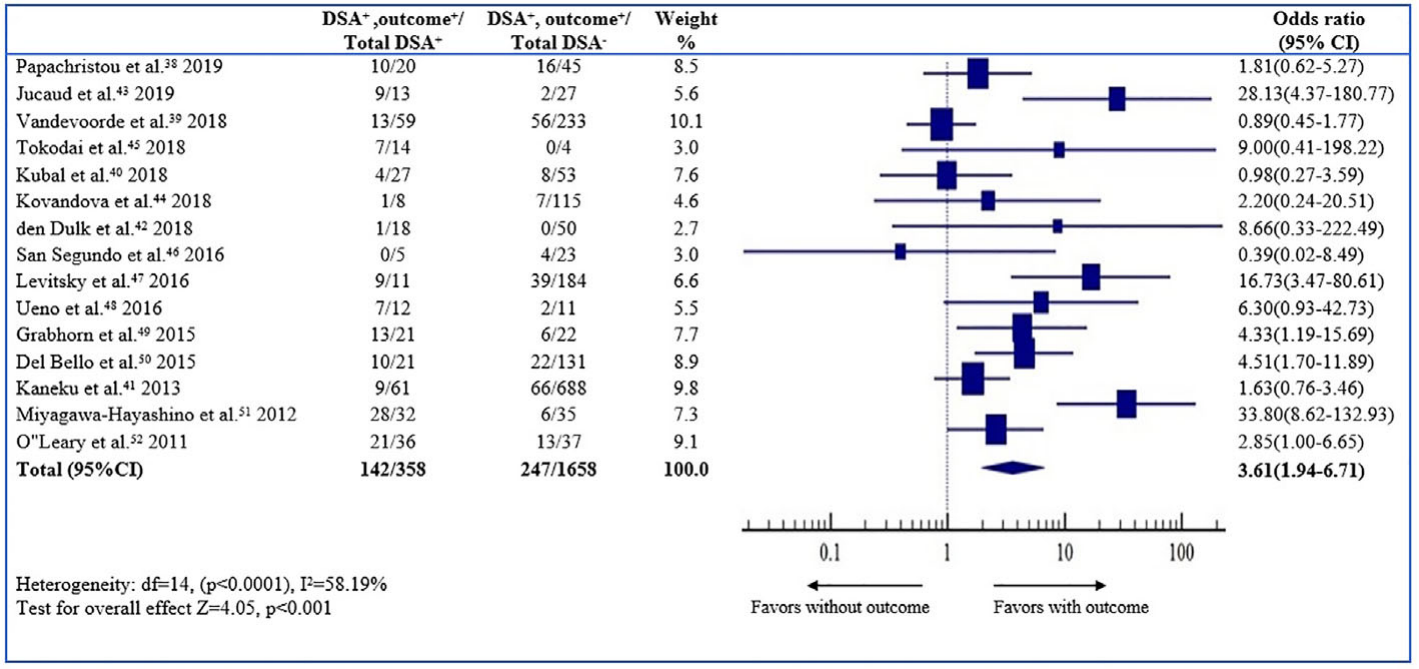
Association between *de novo* DSAs and the risk of overall outcome. Forest plot of comparison of patients with *de novo* DSA vs. those without *de novo* DSA with outcome.

The odds ratio (OR) of the outcome (graft loss or rejection) was 3.61 (95% CI, 1.94–6.71, *P* < 0.001; I^2^ 58.19%) for the dn-DSA-positive patients, which implies a 3.61 times higher graft loss or rejection on long term outcome compared to the dn-DSA-negative patients (**Figure 2**). The result was statistically significant.

#### Risk of Allograft Loss According to *De Novo* Donor Specific Anti-HLA DSA Status

**Figure 3:**
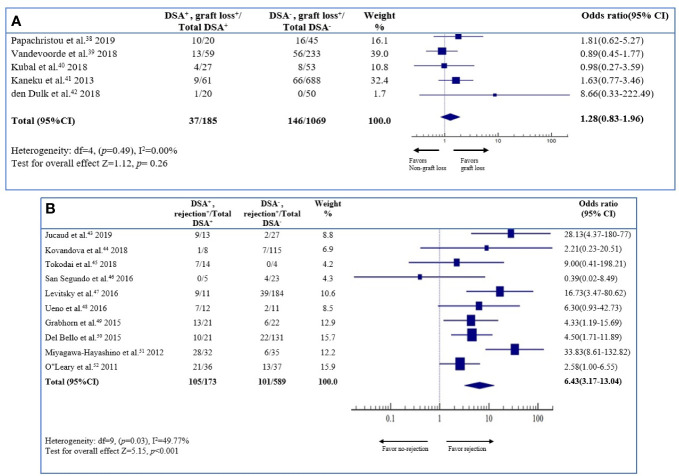
Association between *de novo* DSAs and **(A)** the risk of graft loss, **(B)** rejection. Forest plot of comparison of patients with *de novo* DSA vs. those without *de novo* DSA in each outcome.

Five studies (1,254 patients) were included in the analysis of allograft loss. The odds ratio of the allograft loss was 1.28 (95% CI 0.83–1.96; *P* = 0.26) for the DSA-positive patients, compared to the DSA-negative patients (**Figure 3A**). The result was statistically insignificant. The heterogeneity of the literatures was low (I^2^ 0.00%); therefore, there was no possibility of a publication bias.

#### Risk of Rejection According to *De Novo* Donor Specific Anti-HLA DSA Status

Ten studies (762 patients) were included in the analyses of rejection. The odds ratio of rejection was 6.43 (95% CI 3.17–13.04; *P* < 0.001) for the DSA-positive patients, compared to the DSA-negative patients (**Figure 3B**). The result indicated that dn-DSA positive patients had a 6.43 times higher rejection on long-term outcome compared to the dn-DSA-negative patients. The result was statistically significant. The heterogeneity of the literature was low (I^2^ 49.77%), and there was no possibility of publication bias.

### Subgroup and Sensitivity Analyses

Subgroup analyses were performed on the overall outcome to confirm the consistency of the results and explain some of the heterogeneity found in the main results. Summaries of all different effect sizes for different subgroup analyses are presented in **Table 2**.

**Table 2:** Effect sizes related to different subgroup analyses.

Subgroup and sensitivity analyses for overall outcome (allograft loss and rejection)		Effect size (95% CI)	*P* value	I^2^	Q(df) *P* value
Effect of dn-DSA according to adult or pediatric patients	Pediatric patients studies Adult patients studies	10.20(4.65–22.33)* 2.57(1.38–4.76)	<0.001 0.00	38.81% 51.81%	4.90(3) 0.17 32.17(11) <0.001
Effect of dn-DSA according to type of organ donation	Living donor Deceased donor	15.44(6.32–37.74)* 1.75(0.76–4.02)	<0.001 0.19	0.00% 33.79%	2.65(3) 0.44 6.04(4) 0.19
Effect of dn-DSA according to duration of follow up after transplantation	Follow up ≤3 years Follow up 5 years Follow up >5 years	2.97(1.52–5.78)* 3.64(1.43–9.33)* 3.90(0.93–14.86)	0.00 0.00 0.05	0.00% 54.33% 81.21%	2.06(3) 0.56 8.04(3) 0.03 33.33(6) 0.00
Effect of dn-DSA according to cutoff MFI value	MFI <3,000 MFI ≥3,000	4.28(1.96–9.31) 2.43(0.75–7.83)	<0.001 0.13	59.25% 61.43%	32.43(9) 0.00 10.35(4) 0.31
Effect of dn-DSA according to type of biopsy	Protocol biopsy Indication biopsy	3.55(1.40–9.02) 3.71(1.75–7.79)	0.00 0.00	51.75% 37.00%	19.93(7) <0.001 9.52(6) 0.14
Effect of dn-DSA according to confounding factors		5.11(1.70–15.33)	0.00	57.37%	20.21(7) 0.00
Effect of dn-DSA in studies with high methodological quality		3.53(1.77–7.07)	<0.001	46.11%	22.14(10) 0.00
Center effect		4.44(1.93–10.21)	<0.001	52.33%	24.89(9) 0.00

Effect sizes refer to OR for outcome appearance. *Fixed effects for OR was reported.

#### Effect of dn-DSA According to Adult or Pediatric Patients

Sensitivity analysis restricted to studies with pediatric patients (4 studies; 151 patients) was used to demonstrate consistent results regarding the association between dn-DSA positive and overall outcome with an increased OR to 10.20 as compared to patients with negative dn-DSA (95% CI 4.65–22.33; *P* < 0.001; I^2^ 38.81%) (**Figure S1**). In addition, the heterogeneity across the studies decreased from 58.19 to 38.81%. Eleven studies (1,865 adult patients) were included in the sub-analysis of the overall outcome, which did not differ much from the overall results (OR 2.57; 95% CI 1.38–4.76; *P* = 0.00; I^2^ 51.81%) (**Figure S2**).

#### Effect of dn-DSA According to the Type of Organ Donation

We performed a stratified sensitivity analysis according to the donor type of LT patients for detection of dn-DSA. We found out that patients with LDLT and positive dn-DSA (4 studies, 237 patients) showed increased OR to 15.44 (95% CI 6.32–37.74; *P* < 0.001; I^2^ 0.00%) significantly (**Figure S3**). Hence, 5 studies (614 patients) with the DDLT patients were analyzed with decreased OR from the overall results (OR 1.75; 95% CI 0.76–4.02; *P* = 0.19; I^2^ 33.79%) (**Figure S4**).

#### Effect of dn-DSA According to the Duration of Follow Up Post-Transplantation

Sensitivity analysis restricted to studies (4 studies; 408 patients) with follow up time ≤3 years indicated results regarding the association between positive dn-DSA and the risk of long-term overall outcome, with a pooled OR of 2.97 (95% CI 1.52–5.78; *P* = 0.00; I^2^ 0.00%), and the heterogeneity across the studies decreased from 58.19 to 0.00% (**Figure S5**). Sensitivity analysis restricted to studies (4 studies; 905 patients) with the follow up duration of 5 years demonstrated consistent results regarding the association between positive dn-DSA and the risk of overall outcome, with an increased OR of 3.64 (95% CI 1.43–9.33; *P* = 0.00; I^2^ 54.33) (**Figure S6**). Sensitivity analysis restricted to studies (7 studies; 703 patients) with the follow up time of >5 years demonstrated consistent results regarding the association between positive dn-DSA and the risk of long-term overall outcome, with an increased OR of 3.90 (95% CI 0.93–14.86; *P* = 0.05; I^2^ 81.21) (**Figure S7**).

#### Effect of dn-DSA According to Cutoff MFI Value

We performed a stratified sensitivity analysis according to the cutoff MFI value for detection of dn-DSA. It was confirmed that patients with cutoff MFI value lower than 3,000 (OR 4.28; 95% CI 1.96–9.31; *P* < 0.00; I^2^ 59.25%) positive dn-DSA were significantly associated with an increased risk of overall outcome (**Figure S8**). However, in patients with the cutoff MFI value higher than 3,000 (OR 2.43; 95% CI 0.75–7.83; *P* = 0.13; I^2^ 61.43%) positive dn-DSA remained insignificant (**Figure S9**).

#### Effect of dn-DSA According to the Type of Biopsy

We performed a stratified sensitivity analysis according to detection of patient by protocol biopsy or indication. It was confirmed that in patients with either protocol (OR 3.55; 95% CI 1.40–9.02, *P* = 0.00; I^2^ 51.75%) or indication (OR 3.71; 95% CI 1.75–7.79; *P* = 0.00; I^2^ 37.00%) biopsy, dn-DSAs remained significantly associated with an increased risk of overall outcome (**Figures S10, S11**).

#### Effect of dn-DSA According to the Confounding Factors

Primary analyses were stratified sensitivity analysis according to the lack of any confounding factors such as CMV, viral disease, technical problem, etc. In 8 studies, we found consistent associations between dn-DSA production and risk of the overall outcome in patients without confounding variables (OR 5.11; 95% CI 1.70–15.33; *P* = 0.00; I^2^ 57.37%) (**Figure S12**).

#### Effect of dn-DSA in Studies With High Methodological Quality

Sensitivity analysis restricted to studies with high methodological quality (NOS score > 7) demonstrated consistent results regarding the association between dn-DSA and the risk of a long-term outcome (OR 3.53; 95% CI 1.77–7.07; *P* < 0.001; I^2^ 46.11) (**Figure S13**).

#### Center Effect

After removing the 5 largest cohort studies (in terms of the number of patients included) from the analysis (37, 39, 42, 45, 48), the presence of dn-DSA remained significantly associated with an increased risk of an outcome (OR 4.44; 95% CI 1.93–10.21; *P* < 0.001), and the heterogeneity across the studies decreased from 58.19 to 52.33% (**Figure S14**).

## Discussion

The humoral immune system is a significant barrier to solid organ transplantation due to antibodies against non-self-proteins expressed on transplanted organs (51, 52). In the majority of liver transplant programs, the liver is transplanted without cross-match results (53–58) because it is believed that the liver is tolerant to humoral response (antibody mediated rejection). Recent studies demonstrated that a positive cross-match could increase the risk of early graft loss in liver transplanted patients which can be induced by circulating DSAs (59–62). However, it seems that DSAs are one of the essential parts of tolerance development (63, 64). Since consistent results are lacking, clinical practice has not been significantly changed (65). Therefore, the meta-analysis, as an innovative, quantitative statistical method for medical assessment, might be used for clarification when controversy persists.

This is the first systematic review and meta-analysis in the available literature which investigated the role of dn-DSAs on the graft outcome in LT recipients. In the present meta-analysis, we included 15 studies, with 2016 patients from the USA, Europe, and Japan. The statistical analysis reveals the association of the dn-DSA with long-term outcome in LT recipients. In addition to the overall outcome, we found that dn-DSA was strongly associated with an increased risk of graft rejection without a significant publication bias and with acceptable heterogeneity. As to rejection, dn-DSA positive patients had a 6.43 times higher rejection rate in the long-term follow up compared to the dn-DSA-negative patient. These results may enhance the validity of the findings and their applicability in various therapeutic applications, and transplant programs with different practices, and also support the possibility of a causal effect between dn-DSA and allograft outcome. It is worth mentioning that not all dn-DSA classes are equal in terms of pathogenicity; therefore, they might be associated with different adverse allograft outcomes. In view of the fact that activation of dn-DSA along with complement cascade is a major component of AMR process, new investigations have been useful for better understanding of the role of DSAs and pathophysiology of transplant allograft outcome (66).

The findings of this meta-analysis were confirmed by different subgroup analyses. First, even though adult LT patients were the highest number of patients included in the present meta-analysis, the effects of dn- DSAs on the allograft outcome remained significant in pediatric LT patients with an increase of 10.20 times compared to patients with negative dn-DSA. Second, the LDLT leads to dn-DSA and higher adverse effects on the allograft outcome compared to DDLT. It should be mentioned that due to small number of studies and low sample size in each study, the confidence interval is very wide. Hence, the results should be interpreted with caution and may not be reliable. Third, the same effect was observed regardless of the follow-up duration after transplantation. Fourth, we found similar associations regardless of the lower cutoff MFI value (MFI < 3,000), not high ones (MFI ≥ 3,000). To note, the low range cutoff was the same in all studies, which was 1,000 MFI; however, the result of the MFI ≥3,000 value was insignificant which might be due to different ranges from 3,000–5,000 MFI value between studies. These different cutoffs MFI and technical issues in dn-DSA detection is out of the scope of the present study.

Additionally, our sub-analysis clearly shows that not only protocoled biopsy, but also induction biopsy have the same effect of dn-DSA on the outcome. Indeed, we analyzed the effect of dn-DSA according to the potential of confounding factors such as CMV, viral disease, technical problem, etc., which might also lead to dn-DSA production due to alloantigen exposure. As to the factors associated with DSA, it was reported that CMV infection was correlated with AMR, chronic rejection, and allograft dysfunction in liver transplant (36). Our finding in studies without confounding factors suggests a strong relationship between independent production of dn-DSA and outcome. Despite this overall heterogeneity, when sensitivity analyses were performed including studies with high methodological quality, the heterogeneity decreased from 58.19 to 46.11%. Finally, we performed the center effect on the analysis because larger studies might be an effective factor for the finding associations in primary analyses and could change the overall heterogeneity.

Based on the subgroup analysis, non-optimal statistical power and statistical methodologies used in the studies might elucidate the heterogeneity (I^2^) distinguished in our study. Despite the overall heterogeneity, when the above-mentioned subgroup analyses were performed, the heterogeneity decreased from 58.19% in some sub-analyses. Therefore, the association between dn-DSA and allograft outcome remained significant in different transplant populations, types of organ donation, duration of follow up post-transplantation, high quality mythological studies, and the center effect, thereby reinforcing the study conclusions. However, further research is needed because of low number of studies and heterogeneity higher than 50%.

The results of the present investigation might have an important clinical implication. The overall association represented in this study could increase the validity of using dn-DSAs as a potential prognostic factor in allograft rejection of LT patients. It is known that one of the main problems to improve and develop the overall LT patient outcomes is the lack of reliable, well-informed, and noninvasive biomarkers to predict the allograft outcomes (66). As a result, knowledgeable biomarker such as a dn-DSA can be used for classification of the patients’ outcome, allograft risk, clinical trial design, and as marker endpoints. Accordingly, monitoring of dn-DSA continuously after LT might take steps toward improving liver transplantation by performing necessary action at the right time such as a maximization/minimization immunosuppression (IS) or IS withdrawal eligibility.

According to the literature data, the presence of DSA is associated with post-LT complications including *de novo* autoimmune hepatitis, ductopenia, biliary strictures, accelerated fibrosis, refractory thrombocytopenia and acute liver injury, which can induce combined acute antibody-mediated rejection (AMR), and T-cell-mediated rejection (18, 67). DSA positivity and high MFI are indicative of AMR, which needs more aggressive immunosuppression and according to BANFF classification, positive DSA is mandatory for the diagnosis of AMR (68). Accordingly, characterization of dn-DSAs may also have therapeutic significance, providing opportunities for the treatment and decision for immune suppression (IS) and minimization of specific drugs targeting. It may also help to find out the reason for immunosuppressive treatment and switch the therapy to more aggressive immunosuppression such as plasmapheresis.

This study had some limitations: we first acknowledge the higher population of adult patients compared to pediatric liver transplant patients. We also acknowledge that fewer studies regarding allograft loss were included. Further studies are required to quantify the value of the effect of dn-DSA on the risk of allograft loss. Third, the exact time of dn-DSA detection could not be addressed because the articles included in the meta-analysis did not report them. Fourth, no data were available from Australian, Asian or South American LT populations, which can restrict the extrapolation of our findings to these patient populations. Fifth, differences in the patients’ characteristics that might have an effect on transplantation outcomes, such as donor age, primary diseases, and immunosuppression maintenance, were not reported in most of the studies. Indeed, the difference between the males and females and different etiologies cannot be assessed given the lack of available data. Finally, almost all the included studies were observational and retrospective, so unknown confounding factors may elucidate a part of the residual heterogeneity observed.

In conclusion, the results of this meta-analysis on circulating dn-DSAs demonstrate a significant determinant of long-term allograft outcome in liver transplant rejection, and consideration might be given to a feasible, valuable prognostic biomarker for enhancement of the risk stratification for liver allograft outcome. Our results may encourage the clinicians, health care providers, and health policy makers to monitor dn-DSA in LT recipients.

## Data Availability Statement

The original contributions presented in the study are included in the article/**Supplementary Material**. Further inquiries can be directed to the corresponding author.

## Author Contributions

ZBe: served as primary investigator for the study, helped to design the study, directed data collection, analyzed data and interpretation, created first draft of the manuscript, edited the manuscript. ZBa: analyzed the data and interpreted them. SK and AS: collected the data. BG: senior author, created the project, coordinated data collection, critically revised the work, and edited the manuscript. All authors contributed to the article and approved the submitted version.

## Conflict of Interest

The authors declare that the research was conducted in the absence of any commercial or financial relationships that could be construed as a potential conflict of interest.

## Abbreviations

AMR, antibody-mediated rejection; CI, Confidence interval; DDLT, deceased donor liver transplant; dn-DSA, *de novo* donor-specific HLA antibodies; HLA, Human leukocyte antigen; IS, immune suppression; LDLT, living donor liver transplant; LT, liver transplant; MFI, mean fluorescence intensity; OR, odds ratio; SAB, single antigen bead assay.
