# Supplementary material for: De Novo Donor Specific Antibody and Long-Term Outcome After Liver Transplantation: A Systematic Review and Meta-Analysis
Source: Front Immunol. 2020 Dec 23;11:613128. doi: 10.3389/fimmu.2020.613128 (PMC7786049; doi:10.3389/fimmu.2020.613128)
Supplement: Supplementary file 1 [file DataSheet_1.pdf]

Supporting information

De novo Donor Specific Antibody and Long Term Outcome after Liver Transplantation: A Systematic Review and Meta-analysis

Zahra Beyzaei PhD, Bita Geramizadeh MD*, Zahra Bagheri PhD, Sara Karimzadeh MSc, Alireza Shojazadeh MD

Table of contents

Supplemental Table 1. Search strategy in databases…………………………………………….4

Supplemental Table 2. Methodological quality assessment of included studies……………….13

Supplemental Figure 1. Forest plot for the overall outcome in accordance to de novo DSA groups of pediatric patients……………………………………………………………………….....14

Supplemental Figure 2. Forest plot for the overall outcome in accordance to de novo DSA groups of adult patients………………………………………………………………………………..15

Supplemental Figure 3. Forest plot for the overall outcome in accordance to living donor of LT patients…………………………………………………………………………………………...16

Supplemental Figure 4. Forest plot for the overall outcome in accordance to deceased donor of LT patients……………………………………………………………………………………….17

Supplemental Fig 5. Forest plot for the overall outcome in accordance to de novo DSA groups of follow up time < 3 years post transplantation..…………………………………..……..……..…..18

Supplemental Fig 6. Forest plot for the overall outcome in accordance to de novo DSA groups of follow up time 5 years post transplantation...……………………..……………………………...19

Supplemental Fig 7. Forest plot for the overall outcome in accordance to de novo DSA groups of follow up time > 5 years post transplantation.…………………………………..……………..20

Supplemental Fig 8. Forest plot for the overall outcome in accordance to de novo DSA groups of lower MFI cutoff value…..........………………………………………………….…………..21

Supplemental Figure 9. Forest plot for the overall outcome in accordance to de novo DSA groups of high MFI cutoff value……………………………………………………………………22

Supplemental Figure 10. Forest plot for the overall outcome in accordance to protocol biopsy…………………………………………………………………………………….....23

Supplemental Figure 11. Forest plot for the overall outcome in accordance to indication biopsy...24

Supplemental Figure 12. Forest plot for the overall outcome in accordance to confounding factors……………………………………………………………………………………….25

Supplemental Fig 13. Forest plot for the overall outcome in accordance to de novo DSA groups of high methodological quality………………… ………………………………..…….26

Supplemental Figure 14. Forest plot for the overall outcome in accordance to de novo DSA groups of center effect…………………………………………………………………...27

Supplemental Table 1. Search strategy in databases including MEDLINE/PubMed, EMBASE, Cochrane Library, Scopus and Web of Science Core Collection databases.

**PubMed**	((((((((((((((((hepatic transplant* OR hepatic graft* OR hepatic allotransplant* OR hepatic homotransplant* OR hepatic retransplant* OR hepatic autotransplant* OR hepatic allograft* OR hepatic homograft* OR liver transplant* OR liver graft* OR liver allotransplant* OR liver homotransplant* OR liver retransplant* OR liver autotransplant* OR liver allograft* OR liver homograft*)) OR hepatic allo-transplant*) OR hepatic homo-transplant*) OR hepatic re-transplant*) OR hepatic auto-transplant*) OR hepatic allo-graft*) OR hepatic homo-graft*) OR cadaver liver*) OR liver allo-transplant*) OR liver homo-transplant*) OR liver re-transplant*) OR liver auto-transplant*) OR liver allo-graft*) OR liver homo-graft*)) OR "Liver Transplantation"[Mesh])	76830
	((((((("de novo donor-specific anti-HLA antibodies" OR "complement activating" OR "complement binding" OR DSA OR dsas OR "donor specific" OR antibod* OR "anti-bod*" OR alloantibod* OR "allo-antibod*" OR "donor specific" OR "donor HLA-specific" OR "human leukocyte antigen*" OR "anti-HLA" OR anti hla OR HLA OR "antiHLA DSA" OR "antiHLA DSAs" OR C1q OR C3d OR C4d OR "HLA DSA" OR "HLA DSAs" OR "HLA-A" OR "HLA-B" OR "HLA-DRB1" OR "HLA-DRB3" OR "HLA-DRB345" OR "HLA-DQ" OR "HLA-DP" OR "IgG subclass*" OR "IgG1 subclass*" OR "IgG3 subclass*" OR "IgG4 subclass*" OR "immunoglobulin G1 subclass*" OR "immunoglobulin G3 subclass*" OR "immunoglobulin G4 subclass*" OR "immunoglobulin-G subclass*" OR "single-antigen bead array*"))) OR ((((((("Complement C3d"[Mesh]) OR "HLA-B Antigens"[Mesh]) OR "HLA-DRB1 Chains"[Mesh]) OR "HLA-DRB3 Chains"[Mesh]) OR "HLA-DQ Antigens"[Mesh]) OR "HLA-DP Antigens"[Mesh]) OR "Immunoglobulin G"[Mesh])) OR (((("Complement Activating Enzymes"[Mesh]) OR "Antibodies"[Mesh]) OR "Complement C1q"[Mesh]) OR "complement C4d" [Supplementary Concept])))) OR "HLA-DR Antigens"[Mesh]	1412821
	((((((((((((((Treatment Outcome) OR Graft Rejection) OR Graft Survival) OR reaction*) OR host defense reaction*) OR host donor cell interaction*) OR host graft response*) OR host versus graft reaction*) OR host versus graft syndrome*) OR loss) OR outcome*) OR reject*) OR surviv*)) OR (((("Treatment Outcome"[Mesh]) OR "Graft Rejection"[Mesh]) OR "Graft Survival"[Mesh]) OR "Host vs Graft Reaction"[Mesh])	5763318
	(("meta analysis" OR "Meta-Analysis" OR "systematic review" OR Guideline OR review OR "minireview" OR "mini-review"))	3463780

	(alpaca OR alpacas OR amphibian OR amphibians OR animal OR animals OR antelope OR armadillo OR armadillos OR avian OR baboon OR baboons OR beagle OR beagles OR bee OR bees OR bird OR birds OR bison OR bovine OR buffalo OR buffaloes OR buffalos OR "c elegans" OR "Caenorhabditis elegans" OR camel OR camels OR canine OR canines OR carp OR cats OR cattle OR chick OR chicken OR chickens OR chicks OR chimp OR chimpanze OR chimpanzees OR chimps OR cow OR cows OR "D melanogaster" OR "dairy calf" OR "dairy calves" OR deer OR dog OR dogs OR donkey OR donkeys OR drosophila OR "Drosophila melanogaster" OR duck OR duckling OR ducklings OR ducks OR equid OR equids OR equine OR equines OR feline OR felines OR ferret OR ferrets OR finch OR finches OR fish OR flatworm OR flatworms OR fox OR foxes OR frog OR frogs OR "fruit flies" OR "fruit fly" OR "G mellonella" OR "Galleria mellonella" OR geese OR gerbil OR gerbils OR goat OR goats OR goose OR gorilla OR gorillas OR hamster OR hamsters OR hare OR hares OR heifer OR heifers OR horse OR horses OR insect OR insects OR jellyfish OR kangaroo OR kangaroos OR kitten OR kittens OR lagomorph OR lagomorphs OR lamb OR lambs OR llama OR llamas OR macaque OR macaques OR macaw OR macaws OR marmoset OR marmosets OR mice OR minipig OR minipigs OR mink OR minks OR monkey OR monkeys OR mouse OR mule OR mules OR nematode OR nematodes OR octopus OR octopuses OR orangutan OR "orang-utan" OR orangutans OR "orang-utans" OR oxen OR parrot OR parrots OR pig OR pigeon OR pigeons OR piglet OR piglets OR pigs OR porcine OR primate OR primates OR quail OR rabbit OR rabbits OR rat OR rats OR reptile OR reptiles OR rodent OR rodents OR ruminant OR ruminants OR salmon OR sheep OR shrimp OR slug OR slugs OR swine OR tamarin OR tamarins OR toad OR toads OR trout OR urchin OR urchins OR vole OR voles OR waxworm OR waxworms OR worm OR worms OR xenopus OR "zebra fish" OR zebrafish)	6897856
PubMed A	1 AND 2 AND 3 NOT 4	3412
PubMed B	6 NOT 5	128

**Cochrane**	hepatic transplant* OR hepatic graft* OR hepatic allotransplant* OR hepatic homotransplant* OR hepatic retransplant* OR hepatic autotransplant* OR hepatic allograft* OR hepatic homograft* OR liver transplant* OR liver graft* OR liver allotransplant* OR liver homotransplant* OR liver retransplant* OR liver autotransplant* OR liver allograft* OR liver homograft* OR hepatic allo-transplant* OR hepatic homo-transplant* OR hepatic re-transplant* OR hepatic auto-transplant* OR hepatic allo-graft* OR hepatic homo-graft* OR cadaver	99

	liver* OR liver allo-transplant* OR liver homo-transplant* OR liver re-transplant* OR liver auto-transplant* OR liver allo-graft* OR liver homo-graft*	
	"de novo donor-specific anti-HLA antibodies" OR "complement activating" OR "complement binding" OR DSA OR dsas OR "donor specific" OR antibod* OR "anti-bod*" OR alloantibod* OR "allo-antibod*" OR "donor specific" OR "donor HLA-specific" OR "human leukocyte antigen*" OR "anti-HLA" OR anti hla OR HLA OR "antiHLA DSA" OR "antiHLA DSAs" OR C1q OR C3d OR C4d OR "HLA DSA" OR "HLA DSAs" OR "HLA-A" OR "HLA-B" OR "HLA-DRB1" OR "HLA-DRB3" OR "HLA-DRB345" OR "HLA-DQ" OR "HLA-DP" OR "IgG subclass*" OR "IgG1 subclass*" OR "IgG3 subclass*" OR "IgG4 subclass*" OR "immunoglobulin G1 subclass*" OR "immunoglobulin G3 subclass*" OR "immunoglobulin G4 subclass*" OR "immunoglobulin-G subclass*" OR "single-antigen bead array*"	868
	Treatment Outcome OR Graft Rejection OR Graft Survival OR reaction* OR host defense reaction* OR host donor cell interaction* OR host graft response* OR host versus graft reaction* OR host versus graft syndrome* OR loss OR outcome* OR reject* OR surviv*	6892
	alpaca OR alpacas OR amphibian OR amphibians OR animal OR animals OR antelope OR armadillo OR armadillos OR avian OR baboon OR baboons OR beagle OR beagles OR bee OR bees OR bird OR birds OR bison OR bovine OR buffalo OR buffaloes OR buffalos OR "c elegans" OR "Caenorhabditis elegans" OR camel OR camels OR canine OR canines OR carp OR cats OR cattle OR chick OR chicken OR chickens OR chicks OR chimp OR chimpanze OR chimpanzees OR chimps OR cow OR cows OR "D melanogaster" OR "dairy calf" OR "dairy calves" OR deer OR dog OR dogs OR donkey OR donkeys OR drosophila OR "Drosophila melanogaster" OR duck OR duckling OR ducklings OR ducks OR equid OR equids OR equine OR equines OR feline OR felines OR ferret OR ferrets OR finch OR finches OR fish OR flatworm OR flatworms OR fox OR foxes OR frog OR frogs OR "fruit flies" OR "fruit fly" OR "G mellonella" OR "Galleria mellonella" OR geese OR gerbil OR gerbils OR goat OR goats OR goose OR gorilla OR gorillas OR hamster OR hamsters OR hare OR hares OR heifer OR heifers OR horse OR horses OR insect OR insects OR jellyfish OR kangaroo OR kangaroos OR kitten OR kittens OR lagomorph OR lagomorphs OR lamb OR lambs OR llama OR llamas OR macaque OR macaques OR macaw OR macaws OR marmoset OR marmosets OR mice OR minipig OR minipigs OR mink OR minks OR monkey OR monkeys OR mouse OR mule OR mules OR nematode OR nematodes OR octopus OR octopuses OR orangutan OR "orang-utan" OR orangutans OR "orang-utans" OR oxen OR parrot OR parrots OR pig OR pigeon OR pigeons OR piglet OR piglets OR pigs OR porcine OR primate OR primates OR quail OR rabbit OR rabbits OR rat OR	7318

	rats OR reptile OR reptiles OR rodent OR rodents OR ruminant OR ruminants OR salmon OR sheep OR shrimp OR slug OR slugs OR swine OR tamarin OR tamarins OR toad OR toads OR trout OR urchin OR urchins OR vole OR voles OR waxworm OR waxworms OR worm OR worms OR xenopus OR "zebra fish" OR zebrafish	
	(#1 AND #2 AND #3) NOT #4	541

**Embase**	hepatic AND transplant* OR (hepatic AND graft*) OR (hepatic AND allotransplant*) OR (hepatic AND homotransplant*) OR (hepatic AND retransplant*) OR (hepatic AND autotransplant*) OR (hepatic AND allograft*) OR (hepatic AND homograft*) OR (liver AND transplant*) OR (liver AND graft*) OR (liver AND allotransplant*) OR (liver AND homotransplant*) OR (liver AND retransplant*) OR (liver AND autotransplant*) OR (liver AND allograft*) OR (liver AND homograft*) OR (hepatic AND 'allo transplant*') OR (hepatic AND 'homo transplant*') OR (hepatic AND 're transplant*') OR (hepatic AND 'auto transplant*') OR (hepatic AND 'allo graft*') OR (hepatic AND 'homo graft*') OR (cadaver AND liver*) OR (liver AND 'allo transplant*') OR (liver AND 'homo transplant*') OR (liver AND 're transplant*') OR (liver AND 'auto transplant*') OR (liver AND 'allo graft*') OR (liver AND 'homo graft*')	213553
	'de novo donor-specific anti-HLA antibodies' OR 'complement activating' OR 'complement binding'/exp OR 'complement binding' OR dsa OR dsas OR antibod* OR 'anti-bod*' OR alloantibod* OR 'allo-antibod*' OR 'donor specific' OR 'donor hla-specific' OR 'human leukocyte antigen*' OR 'anti-hla' OR 'anti hla' OR (anti AND ('hla'/exp OR hla)) OR 'hla'/exp OR hla OR 'antihla dsa' OR 'antihla dsas' OR 'c1q'/exp OR c1q OR 'c3d'/exp OR c3d OR 'c4d'/exp OR c4d OR 'hla dsa' OR 'hla dsas' OR 'hla-a' OR 'hla-b' OR 'hla-drb1'/exp OR 'hla-drb1' OR 'hla-drb3'/exp OR 'hla-drb3' OR 'hla-drb345' OR 'hla-dq' OR 'hla-dp' OR 'igg subclass*' OR 'igg1 subclass*' OR 'igg3 subclass*' OR 'igg4 subclass*' OR 'immunoglobulin g1 subclass*' OR 'immunoglobulin g3 subclass*' OR 'immunoglobulin g4 subclass*' OR 'immunoglobulin-g subclass*' OR 'single-antigen bead array*'	1628371
	'treatment outcome'/exp OR 'treatment outcome' OR (('treatment'/exp OR treatment) AND ('outcome'/exp OR outcome)) OR 'graft rejection'/exp OR 'graft rejection' OR (('graft'/exp OR graft) AND ('rejection'/exp OR rejection)) OR 'graft survival'/exp OR 'graft survival' OR (('graft'/exp OR	8983446

	graft) AND ('survival'/exp OR survival)) OR reaction* OR 'host defense'/exp OR 'host defense' OR (('host'/exp OR host) AND ('defense'/exp OR defense) AND reaction*) OR 'host donor cell' OR (('host'/exp OR host) AND ('donor'/exp OR donor) AND ('cell'/exp OR cell) AND interaction*) OR 'host graft' OR (('host'/exp OR host) AND ('graft'/exp OR graft) AND response*) OR (('host'/exp OR host) AND versus AND ('graft'/exp OR graft) AND reaction*) OR 'host versus graft' OR (('host'/exp OR host) AND versus AND ('graft'/exp OR graft) AND syndrome*) OR loss OR outcome* OR reject* OR surviv*	
	alpaca OR alpacas OR amphibian OR amphibians OR animal OR animals OR antelope OR armadillo OR armadillos OR avian OR baboon OR baboons OR beagle OR beagles OR bee OR bees OR bird OR birds OR bison OR bovine OR buffalo OR buffaloes OR buffalos OR "c elegans" OR "Caenorhabditis elegans" OR camel OR camels OR canine OR canines OR carp OR cats OR cattle OR chick OR chicken OR chickens OR chicks OR chimp OR chimpanze OR chimpanzees OR chimps OR cow OR cows OR "D melanogaster" OR "dairy calf" OR "dairy calves" OR deer OR dog OR dogs OR donkey OR donkeys OR drosophila OR "Drosophila melanogaster" OR duck OR duckling OR ducklings OR ducks OR equid OR equids OR equine OR equines OR feline OR felines OR ferret OR ferrets OR finch OR finches OR fish OR flatworm OR flatworms OR fox OR foxes OR frog OR frogs OR "fruit flies" OR "fruit fly" OR "G mellonella" OR "Galleria mellonella" OR geese OR gerbil OR gerbils OR goat OR goats OR goose OR gorilla OR gorillas OR hamster OR hamsters OR hare OR hares OR heifer OR heifers OR horse OR horses OR insect OR insects OR jellyfish OR kangaroo OR kangaroos OR kitten OR kittens OR lagomorph OR lagomorphs OR lamb OR lambs OR llama OR llamas OR macaque OR macaques OR macaw OR macaws OR marmoset OR marmosets OR mice OR minipig OR minipigs OR mink OR minks OR monkey OR monkeys OR mouse OR mule OR mules OR nematode OR nematodes OR octopus OR octopuses OR orangutan OR "orang-utan" OR orangutans OR "orang-utans" OR oxen OR parrot OR parrots OR pig OR pigeon OR pigeons OR piglet OR piglets OR pigs OR porcine OR primate OR primates OR quail OR rabbit OR rabbits OR rat OR rats OR reptile OR reptiles OR rodent OR rodents OR ruminant OR ruminants OR salmon OR sheep OR shrimp OR slug OR slugs OR swine OR tamarin OR tamarins OR toad OR toads OR trout OR urchin OR urchins OR vole OR voles OR waxworm OR waxworms OR worm OR worms OR xenopus OR "zebra fish" OR zebrafish	27704895
	(#1 AND #2 AND #3) NOT #4	728

**Scopus**	((TITLE-ABS-KEY (hepatic AND transplant* OR hepatic AND graft* OR hepatic AND allotransplant* OR hepatic AND homotransplant* OR hepatic AND	55444

	retransplant* OR hepatic AND autotransplant* OR hepatic AND allograft* OR hepatic AND homograft*) OR TITLE-ABS-KEY (liver AND transplant* OR liver AND graft*))) OR ((TITLE-ABS-KEY (liver AND allotransplant* OR liver AND homotransplant* OR liver AND retransplant* OR liver AND autotransplant* OR liver AND allograft* OR liver AND homograft*) OR TITLE-ABS-KEY (hepatic AND allo-transplant* OR hepatic AND homo-transplant* OR hepatic AND re-transplant* OR hepatic AND auto-transplant*) OR TITLE-ABS-KEY (hepatic AND allo-graft* OR hepatic AND homo-graft* OR cadaver AND liver* OR liver AND allo-transplant* OR liver AND homo-transplant* OR liver AND re-transplant* OR liver AND auto-transplant* OR liver AND allo-graft* OR liver AND homo-graft*)))	
	(TITLE-ABS-KEY (" de novo donor-specific anti-HLA antibodies ") OR TITLE-ABS-KEY ("complement activating") OR TITLE-ABS-KEY ("complement binding") OR TITLE-ABS-KEY (dsa OR dsas) OR TITLE-ABS-KEY ("donor specific") OR TITLE-ABS-KEY (antibod* OR "anti-bod*") OR TITLE-ABS-KEY (alloantibod*) OR TITLE-ABS-KEY ("allo-antibod*") OR TITLE-ABS-KEY ("donor specific" OR "donor HLA-specific" OR "human leukocyte antigen*") OR TITLE-ABS-KEY ("anti-HLA" OR anti AND hla OR hla OR "antiHLA DSA") OR TITLE-ABS-KEY ("antiHLA DSAs") OR TITLE-ABS-KEY (c1q OR c3d OR c4d) OR TITLE-ABS-KEY ("HLA DSA" OR "HLA DSAs" OR "HLA-A") OR TITLE-ABS-KEY ("HLA-B" OR "HLA-DRB1" OR "HLA-DRB3" OR "HLA-DRB345") OR TITLE-ABS-KEY ("HLA-DQ" OR "HLA-DP" OR "IgG subclass*") OR TITLE-ABS-KEY ("IgG1 subclass*" OR "IgG3 subclass*" OR "IgG4 subclass*" OR "immunoglobulin G1 subclass*") OR TITLE-ABS-KEY ("immunoglobulin G3 subclass*" OR "immunoglobulin G4 subclass*" OR "immunoglobulin-G subclass*" OR "single-antigen bead array*"))	1536301
	(TITLE-ABS-KEY (treatment AND outcome) OR TITLE-ABS-KEY (graft AND rejection) OR TITLE-ABS-KEY (graft AND survival) OR TITLE-ABS-KEY (reaction*) OR TITLE-ABS-KEY (host AND defense AND reaction*) OR TITLE-ABS-KEY (host AND donor AND cell AND interaction*) OR TITLE-ABS-KEY (host AND graft AND response*) OR TITLE-ABS-KEY (host AND versus AND graft AND reaction*) OR TITLE-ABS-KEY (host AND versus AND graft AND syndrome*) OR TITLE-ABS-	11364765

	KEY (loss) OR TITLE-ABS-KEY (outcome*) OR TITLE-ABS-KEY (reject*) OR TITLE-ABS-KEY (surviv*))	
	(TITLE-ABS-KEY (alpaca OR alpacas OR amphibian OR amphibians OR animal OR animals OR antelope OR armadillo OR armadillos OR avian OR baboon OR baboons) OR TITLE-ABS-KEY (beagle OR beagles OR bee OR bees OR bird OR birds OR bison OR bovine OR buffalo OR buffaloes OR buffalos) OR TITLE-ABS-KEY ("c elegans" OR "Caenorhabditis elegans" OR camel OR camels OR canine OR canines OR carp OR cats) OR TITLE-ABS-KEY (cattle OR chick OR chicken OR chickens OR chicks OR chimp OR chimpanze OR chimpanzees OR chimps OR cow OR cows OR "D melanogaster") OR TITLE-ABS-KEY ("dairy calf" OR "dairy calves" OR deer OR dog OR dogs OR donkey OR donkeys OR drosophila) OR TITLE-ABS-KEY ("Drosophila melanogaster" OR duck OR duckling OR ducklings OR ducks OR equid OR equids OR equine OR equines OR feline OR felines OR ferret OR ferrets OR finch OR finches) OR TITLE-ABS-KEY (fish OR flatworm OR flatworms OR fox OR foxes OR frog OR frogs OR "fruit flies" OR "fruit fly" OR "G mellonella" OR "Galleria mellonella") OR TITLE-ABS-KEY (geese OR gerbil OR gerbils OR goat OR goats OR goose OR gorilla OR gorillas OR hamster OR hamsters OR hare OR hares OR heifer OR heifers OR horse OR horses OR insect OR insects) OR TITLE-ABS-KEY (jellyfish OR kangaroo OR kangaroos OR kitten OR kittens OR lagomorph OR lagomorphs OR lamb OR lambs OR llama OR llamas OR macaque) OR TITLE-ABS-KEY (macaques OR macaw OR macaws OR marmoset OR marmosets OR mice OR minipig OR minipigs OR mink OR minks OR monkey OR monkeys OR mouse OR mule OR mules OR nematode) OR TITLE-ABS-KEY (nematodes OR octopus OR octopuses OR orangutan OR "orang-utan" OR orangutans OR "orang-utans" OR oxen OR parrot OR parrots OR pig OR pigeon OR pigeons OR piglet OR piglets) OR TITLE-ABS-KEY (pigs OR porcine OR primate OR primates OR quail OR rabbit OR rabbits OR rat OR rats OR reptile OR reptiles OR rodent OR rodents OR ruminant OR ruminants) OR TITLE-ABS-KEY (salmon OR sheep OR shrimp OR slug OR slugs OR swine OR tamarin OR tamarins OR toad OR toads OR trout OR urchin OR urchins) OR TITLE-ABS-KEY (vole OR voles OR waxworm OR waxworms OR worm OR worms OR xenopus OR "zebra fish" OR zebrafish))	9,375,077

	(#1 AND #2 AND #3) NOT #4	23

**Web of Science**	**TOPIC:** (hepatic transplant* OR hepatic graft* OR hepatic allotransplant* OR hepatic homotransplant* OR hepatic retransplant* OR hepatic autotransplant* OR hepatic allograft* OR hepatic homograft* OR liver transplant*) *OR* **TOPIC:** (liver graft* OR liver allotransplant* OR liver homotransplant* OR liver retransplant* OR liver autotransplant* OR liver allograft* OR liver homograft*) *OR* **TOPIC:** (hepatic allo-transplant* OR hepatic homo-transplant* OR hepatic re-transplant* OR hepatic auto-transplant* OR hepatic allo-graft* OR hepatic homo-graft* OR cadaver liver* OR liver allo-transplant*) *OR* **TOPIC:** (liver homo-transplant* OR liver re-transplant* OR liver auto-transplant* OR liver allo-graft* OR liver homo-graft*)	135595
	**TOPIC:** ("complement activating" OR "complement binding" OR DSA OR dsas OR "donor specific" OR antibod* OR "anti-bod*" OR alloantibod* OR "allo-antibod*" OR "donor specific" OR "donor HLA-specific" OR "human leukocyte antigen*") *OR* **TOPIC:** ("anti-HLA" OR anti hla OR HLA OR "antiHLA DSA" OR "antiHLA DSAs" OR C1q OR C3d OR C4d OR "HLA DSA" OR "HLA DSAs" OR "HLA-A" OR "HLA-B" OR "HLA-DRB1" OR "HLA-DRB3") *OR* **TOPIC:** ("HLA-DRB345" OR "HLA-DQ" OR "HLA-DP" OR "IgG subclass*" OR "IgG1 subclass*" OR "IgG3 subclass*" OR "IgG4 subclass*" OR "immunoglobulin G1 subclass*" OR "immunoglobulin G3 subclass*") *OR* **TOPIC:** ("immunoglobulin G4 subclass*" OR "immunoglobulin-G subclass*" OR "single-antigen bead array*")	1,054,589
	**TOPIC:** (Treatment Outcome OR Graft Rejection OR Graft Survival OR reaction* OR host defense reaction* OR host donor cell interaction* OR host graft response* OR host versus graft reaction* OR host versus graft syndrome* OR loss OR outcome* OR reject* OR surviv*)	7,359,469
	((((**TOPIC:** ((((((((((((((((((((alpaca OR alpacas) OR amphibian) OR amphibians) OR animal) OR animals) OR antelope) OR armadillo) OR armadillos) OR avian) OR baboon) OR baboons) OR beagle) OR beagles) OR bee) OR bees) OR bird) OR birds) OR bison) OR bovine) OR buffalo) OR **TOPIC:** (((((((((((((((((((buffaloes OR buffalos) OR "c elegans") OR "Caenorhabditis elegans") OR camel) OR camels) OR canine) OR canines) OR carp) OR cats) OR cattle) OR chick) OR chicken) OR chickens) OR chicks) OR chimp) OR	7,071,280

	**chimpanzee**) OR chimpanzees) OR chimps) OR cow)) OR **TOPIC:** (((((((((((((((((((((((((((((((((((((((cows OR "D melanogaster") OR "dairy calf") OR "dairy calves") OR deer) OR dog) OR dogs) OR donkey) OR donkeys) OR drosophila) OR "Drosophila melanogaster") OR duck) OR duckling) OR ducklings) OR ducks) OR equid) OR equids) OR equine) OR equines) OR feline) OR felines) OR ferret) OR ferrets) OR finch) OR finches) OR fish) OR flatworm) OR flatworms) OR fox) OR foxes) OR frog) OR frogs) OR "fruit flies") OR "fruit fly") OR "G mellonella") OR "Galleria mellonella") OR geese) OR gerbil) OR gerbils)) OR **TOPIC:** ((goat OR goats) OR goose) OR gorilla) OR gorillas) OR hamster) OR hamsters) OR hare) OR hares) OR heifer) OR heifers) OR horse) OR horses) OR insect) OR insects) OR jellyfish) OR kangaroo) OR kangaroos) OR kitten) OR kittens) OR lagomorph) OR lagomorphs) OR lamb) OR lambs) OR llama) OR llamas) OR macaque) OR macaques) OR macaw) OR macaws) OR marmoset) OR marmosets) OR mice) OR minipig) OR minipigs) OR mink) OR minks) OR monkey) OR monkeys) OR mouse) OR mule) OR mules) OR nematode) OR nematodes) OR octopus) OR octopuses) OR orangutan) OR "orang-utan")) OR **TOPIC:** (((orangutans OR "orang-utans") OR oxen) OR parrot) OR parrots) OR pig) OR pigeon) OR pigeons) OR piglet) OR piglets) OR pigs) OR porcine) OR primate) OR primates) OR quail) OR rabbit) OR rabbits) OR rat) OR rats) OR reptile) OR reptiles) OR rodent) OR rodents) OR ruminant) OR ruminants) OR salmon) OR sheep) OR shrimp) OR slug) OR slugs) OR swine) OR tamarin) OR tamarins) OR toad) OR toads) OR trout) OR urchin) OR urchins) OR vole) OR voles) OR **waxworms**) OR **waxworks**) OR worm) OR worms) OR xenopus) OR "zebra fish") OR zebrafish))	
	(#1 AND #2 AND #3) NOT #4	3905

Supplemental Table 2. Methodological quality assessment of included studies. New Castle-Ottawa Scale evaluation of the observational studies included in the meta-analysis (n=15), stratified by year of publication.

Study	Type of study	Selection	Comparability	Outcome	Sum	Bias item(s)
**Maximum score**		********	******	*******	**9**	
Papachristou-2019	cohort	***	*	***	7	Assessment of outcome
Jucaud-2019	cohort	***	*	**	6	Ascertainment of exposure, Assessment of outcome
Vandevoorde-2018	cross-sectional	****	**	***	9	
Tokodai-2018	cohort	***	*	***	7	Selection of the non-exposed cohort
Kubal-2018	cohort	****	*	***	8	
Kovandova-2018	cross-sectional	****	**	***	9	
Den-dulk-2017	case-control	****	*	***	8	
San Segundo-2016	cross-sectional	***	**	*	6	Ascertainment of exposure, Assessment of outcome
Levitsky-2016	cohort	****	*	***	8	
Ueno-2016	cohort	***	*	***	8	Ascertainment of exposure
Grabhorn-2015	cohort	****	**	***	9	
Del Bello-2015	cohort	****	**	***	9	
Kaneku-2013	cohort	****	**	***	9	
Miyagawa-Hayashino-2012	cohort	****	**	**	8	Follow up (results of dn-DSA 12 patients were missed)
O-Leary-2011	cohort	****	*	***	8	

	DSA$^+$, outcome$^+$/Total DSA$^+$	DSA$^-$, outcome$^+$/Total DSA$^-$	Weight %	Odds ratio(95% CI)
Tokodai et al. 2018	7/14	0/4	6.9	9.00(0.41-198.22)
Ueno et al. 2016	7/12	2/11	18.0	6.30(0.93-42.73)
Grabhorn et al. 2015	13/21	6/22	39.8	4.33(1.19-15.69)
Miyagawa-Hayashino et al. 2012	28/32	6/35	35.3	33.80(8.62-132.93)
**Total (95%CI)**	**55/79**	**14/72**	**100.0**	**10.20(4.65-22.33)**

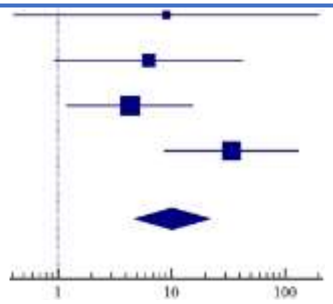

Heterogeneity: df=3, (p=0.45), I^2=38.81%
Test for overall effect Z=5.81, p<0.001

Supplemental Fig 1. Forest plot for the overall outcome in accordance to de novo DSA groups of pediatric patients. The odds ratio (OR) of long-term outcome for the de novo DSA groups. The blue boxes represent the weight of the study, and lines represent the 95% CI for individual studies. The blue diamond at the end represents the pooled OR. Fixed effects for OR was reported.

	DSA⁺, outcome⁺/Total DSA⁺	DSA⁻, outcome⁺/Total DSA⁻	Weight %	Odds ratio(95% CI)
Papachristou et al. 2019	10/20	16/45	8.9	1.81(0.62-5.27)
Jucaud et al. 2019	9/13	2/27	6.0	28.13(4.37-180.77)
Vandevoorde et al. 2018	13/59	56/233	10.4	0.89(0.45-1.77)
Kubal et al. 2018	4/27	8/53	7.9	0.98(0.27-3.59)
Kovandova et al. 2018	1/8	7/115	4.9	2.20(0.24-20.51)
den Dulk et al. 2018	1/18	0/50	3.0	8.66(0.33-222.49)
San Segundo et al. 2016	0/5	4/23	3.2	0.39(0.02-8.49)
Levitsky et al. 2016	9/11	39/184	6.9	16.73(3.47-80.61)
Del Bello et al. 2015	10/21	22/131	9.3	4.51(1.70-11.89)
Kaneku et al. 2013	9/61	66/688	10.1	1.63(0.76-3.46)
O"Leary et al. 2011	21/36	13/37	9.4	2.85(1.00-6.65)
**Total (95%CI)**	**87/279**	**233/1586**	**100.0**	**2.57(1.38-4.76)**

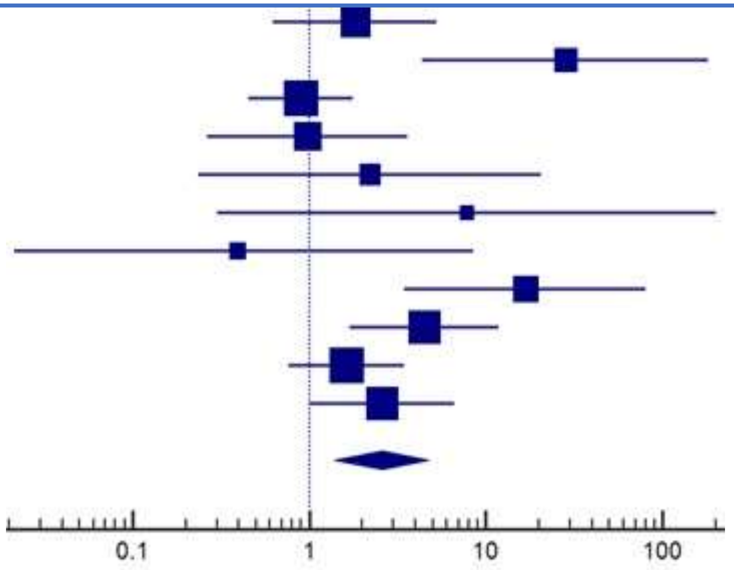

Heterogeneity: df=10, (*p*=0.00), I^2=51.81%
Test for overall effect Z=2.98, *p*=0.00

Supplemental Fig 2. Forest plot for the overall outcome in accordance to de novo DSA groups of adult patients. The odds ratio (OR) of long-term outcome for the de novo DSA groups. The blue boxes represent the weight of the study, and lines represent the 95% CI for individual studies. The blue diamond at the end represents the pooled OR.

	DSA$^+$, outcome$^+$/Total DSA$^+$	DSA$^-$, outcome$^+$/Total DSA$^-$	Weight %	Odds ratio (95% CI)
Tokodai et al. 2018	7/14	0/4	8.3	9.00(0.41-198.22)
Levitsky et al. 2016	5/7	18/122	26.9	16.73(3.47-80.61)
Ueno et al. 2016	7/12	2/11	22.4	6.30(0.93-42.73)
Miyagawa-Hayashino et al. 2012	28/32	6/35	42.4	33.80(8.62-132.93)
**Total (95%CI)**	**47/65**	**26/172**	**100.0**	**15.44(6.32-37.74)**

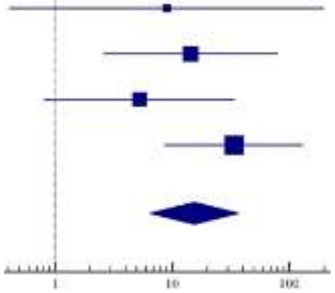

Heterogeneity: df=3, (p=0.44), I^2=0.00%
Test for overall effect Z=6.00, p<0.00

Supplemental Fig 3. Forest plot for the overall outcome in accordance to living donor of LT patients. The blue boxes represent the weight of the study, and lines represent the 95% CI for individual studies. The blue diamond at the end represents the pooled OR. Fixed effects for OR was reported.

	DSA$^+$, outcome$^+$/Total DSA$^+$	DSA$^-$, outcome$^+$/Total DSA$^-$	Weight %	Odds ratio(95% CI)
Papachristou et al. 2019	10/20	16/45	31.1	1.81(0.62-5.27)
Vandevoorde et al. 2018	13/59	56/233	44.4	0.89(0.45-1.77)
den Dulk et al. 2018	1/18	0/50	6.0	8.66(0.33-222.49)
Kovandova et al. 2018	1/8	7/115	11.5	2.20(0.24-20.51)
Levitsky et al. 2016	4/4	21/62	7.0	16.73(3.47-80.61)
**Total (95%CI)**	**29/109**	**100/505**	**100.0**	**1.75(0.76-4.02)**

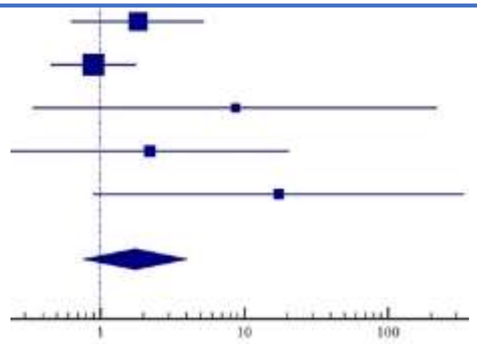

Heterogeneity: df=4, (p=0.19), I^2=33.79%
Test for overall effect Z=1.30, p=0.19

Supplemental Fig 4. Forest plot for the overall outcome in accordance to deceased donor of LT patients. The blue boxes represent the weight of the study, and lines represent the 95% CI for individual studies. The blue diamond at the end represents the pooled OR. Fixed effects for OR was reported.

	DSA$^+$, outcome$^+$/Total DSA$^+$	DSA$^-$, outcome$^+$/Total DSA$^-$	Weight %	Odds ratio(95% CI)
Papachristou et al. 2019	10/20	16/45	39.3	1.81(0.62-5.27)
Del Bello et al. 2015	10/21	22/131	47.5	4.51(1.70-11.89)
den Dulk et al. 2018	1/18	0/50	4.2	8.66(0.33-222.49)
Kovandova et al. 2018	1/8	7/115	9.0	2.20(0.24-20.51)
**Total (95%CI)**	**22/67**	**45/341**	**100.0**	**2.97(1.52-5.78)**

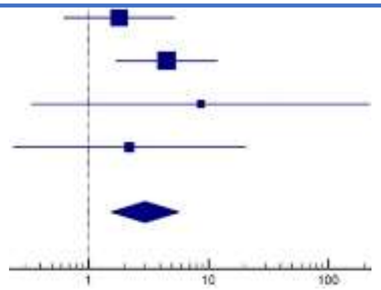

Heterogeneity: df=3, (p=0.50), I^2=0.00%
Test for overall effect Z=3.95, p=0.00

Supplemental Fig 5. Forest plot for the overall outcome in accordance to de novo DSA groups of follow up time < 3 years post transplantation. The odds ratio (OR) of long-term outcome for the de novo DSA groups. The blue boxes represent the weight of the study, and lines represent the 95% CI for individual studies. The blue diamond at the end represents the pooled OR. Fixed effects for OR was reported.

	DSA$^+$, outcome$^+$/Total DSA$^+$	DSA$^-$, outcome$^+$/Total DSA$^-$	Weight %	Odds ratio(95% CI)
Jucaud et al. 2019	9/13	2/27	7.6	28.13(4.37-180.77)
Grabhorn et al. 2015	13/21	6/22	16.0	4.33(1.19-15.69)
Kaneku et al. 2013	9/61	66/688	46.8	1.63(0.76-3.46)
O"Leary et al. 2011	21/36	13/37	29.6	2.85(1.00-6.65)
**Total (95%CI)**	**52/131**	**87/774**	**100.0**	**3.64(1.43-9.33)**

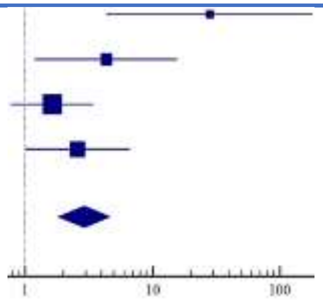

Heterogeneity: df=3, (p=0.03), I^2=54.33%

Test for overall effect Z=4.36, p=0.00

Supplemental Fig 6. Forest plot for the overall outcome in accordance to de novo DSA groups of follow up time 5 years post transplantation. The odds ratio (OR) of long-term outcome for the de novo DSA groups. The blue boxes represent the weight of the study, and lines represent the 95% CI for individual studies. The blue diamond at the end represents the pooled OR.

	DSA$^+$, outcome$^+$/Total DSA$^+$	DSA$^-$, outcome$^+$/Total DSA$^-$	Weight %	Odds ratio (95% CI)
Vandevoorde et al. 2018	13/59	56/233	18.5	0.89(0.45-1.77)
Tokodai et al. 2018	7/14	0/4	9.7	9.00(0.41-198.22)
Kubal et al. 2018	4/27	8/53	16.5	0.98(0.27-3.59)
San Segundo et al. 2016	0/5	4/23	9.7	0.39(0.02-8.49)
Levitsky et al. 2016	4/4	21/62	15.4	16.73(3.47-80.61)
Ueno et al. 2016	7/12	2/11	14.0	6.30(0.93-42.73)
Miyagawa-Hayashino et al. 2012	28/32	6/35	16.2	33.80(8.62-132.93)
**Total (95%CI)**	**68/160**	**115/543**	**100.0**	**3.9(0.93-14.86)**

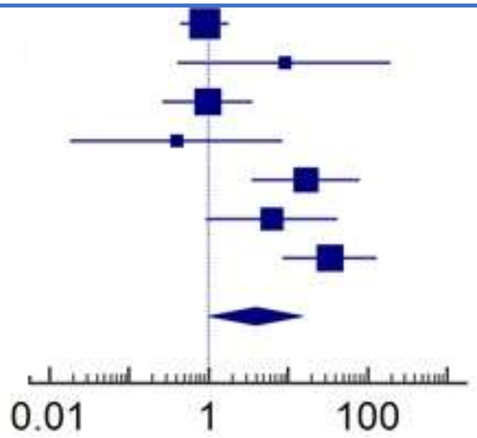

Heterogeneity: df=6, (p=0.00), I^2=81.21%
Test for overall effect Z=1.95, p=0.05

Supplemental Fig 7. Forest plot for the overall outcome in accordance to de novo DSA groups of follow up time > 5 years post transplantation. odds ratio (OR) of long-term outcome for the de novo DSA groups. The blue boxes represent the weight of the study, and lines represent the 95% CI for individual studies. The blue diamond at the end represents the pooled OR.

	DSA$^+$, outcome$^+$/Total DSA$^+$	DSA$^-$, outcome$^+$/Total DSA$^-$	Weight %	Odds ratio (95% CI)
Papachristou et al. 2019	10/20	16/45	12.0	1.81(0.62-5.27)
Jucaud et al. 2018	9/13	2/27	8.2	28.13(4.37-180.77)
Vandevoorde et al. 2018	13/59	56/233	13.9	0.89(0.45-1.77)
Tokodai et al. 2018	7/14	0/4	4.5	9.00(0.41-198.22)
Kovandova et al. 2018	1/8	7/115	6.8	2.20(0.24-20.51)
Ueno et al. 2016	7/12	2/11	8.1	6.30(0.93-42.73)
Grabhorn et al. 2015	13/21	6/22	10.9	4.33(1.19-15.69)
Del Bello et al. 2015	10/21	22/131	12.5	4.51(1.70-11.89)
Miyagawa-Hayashino et al. 2012	28/32	6/35	10.5	33.81(8.62-132.93)
O"Leary et al. 2011	21/36	13/37	12.6	2.85(1.00-6.65)
**Total (95%CI)**	**119/237**	**130/669**	**100.0**	**4.28(1.96-9.31)**

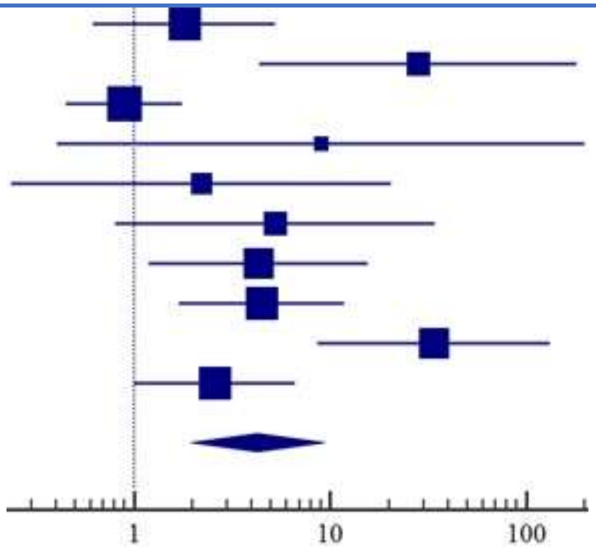

Heterogeneity: df=9, (p<0.0001), I^2=59.25%
Test for overall effect Z=3.6, p<0.001

Supplemental Fig 8. Forest plot for the overall outcome in accordance to de novo DSA groups of lower MFI cutoff value. The odds ratio (OR) of long-term outcome for the de novo DSA groups. The blue boxes represent the weight of the study, and lines represent the 95% CI for individual studies. The blue diamond at the end represents the pooled OR.

	DSA^{+}, outcome^{+}/Total DSA^{+}	DSA^{-}, outcome^{+}/Total DSA^{-}	Weight %	Odds ratio (95% CI)
den Dulk et al. 2018	1/18	0/50	9.6	8.66(0.33-222.49)
Kubal et al. 2018	4/27	8/53	25.5	0.98(0.27-3.59)
Levitsky et al. 2016	9/11	39/184	22.3	16.73(3.47-80.61)
San Segundo et al. 2016	0/5	4/23	10.4	0.39(0.02-8.49)
Kaneku et al. 2013	9/61	66/688	32.2	1.63(0.76-3.46)
**Total (95%CI)**	**23/122**	**117/998**	**100.0**	**2.43(0.75-7.83)**

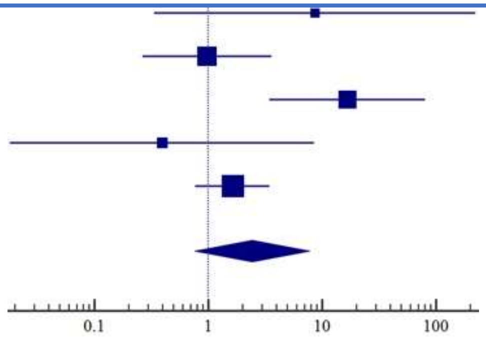

Heterogeneity: df=4, (p=0.31), I^2=61.43%
Test for overall effect Z=1.45, p=0.13

Supplemental Fig 9. **Forest plot for the overall outcome in accordance to de novo DSA groups of high MFI cutoff value.** The odds ratio (OR) of long-term outcome for the de novo DSA groups. The blue boxes represent the weight of the study, and lines represent the 95% CI for individual studies. The blue diamond at the end represents the pooled OR.

	DSA^{+}, outcome^{+}/Total DSA^{+}	DSA^{-}, outcome^{+}/Total DSA^{-}	Weight %	Odds ratio(95% CI)
Kovandova et al. 2018	1/8	7/115	8.7	2.20(0.24-20.51)
Vandevoorde et al. 2018	13/59	56/233	15.8	0.89(0.45-1.77)
Kubal et al. 2018	4/27	8/53	12.9	0.98(0.27-3.59)
Levitsky et al. 2016	9/11	39/184	11.6	16.73(3.47-80.61)
Ueno et al. 2016	7/12	2/11	10.0	6.30(0.93-42.73)
Grabhorn et al. 2015	13/21	6/22	12.9	4.33(1.19-15.69)
Kaneku et al. 2013	9/61	66/688	15.5	1.63(0.76-3.46)
Miyagawa-Hayashino et al. 2012	28/32	6/35	12.6	33.80(8.62-132.93)
**Total (95%CI)**	**84/231**	**190/1341**	**100.0**	**3.55 (1.40-9.02)**

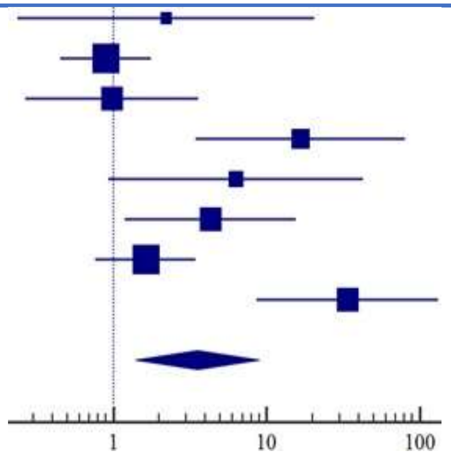

Heterogeneity: df=7, (p<0.001), I^2=51.75%
Test for overall effect Z=2.66, p=0.00

Supplemental Fig 10. Forest plot for the overall outcome in accordance to protocol biopsy. The odds ratio (OR) of long-term outcome for the de novo DSA groups. The blue boxes represent the weight of the study, and lines represent the 95% CI for individual studies. The blue diamond at the end represents the pooled OR.

	DSA⁺, outcome⁺/Total DSA⁺	DSA⁻, outcome⁺/Total DSA⁻	Weight %	Odds ratio(95% CI)
Papachristou et al. 2019	10/20	16/45	22.9	1.81(0.62-5.27)
Jucaud et al. 2019	9/13	2/27	11.7	28.13(4.37-180.77)
Tokodai et al. 2018	7/14	0/4	5.1	9.00(0.41-198.22)
den Dulk et al. 2018	1/18	0/50	4.7	8.66(0.33-222.49)
San Segundo et al. 2016	0/5	4/23	5.2	0.39(0.02-8.49)
Del Bello et al. 2015	10/21	22/131	24.9	4.51(1.70-11.89)
O"Leary et al. 2011	21/36	13/37	25.5	2.85(1.00-6.65)
**Total (95%CI)**	**58/127**	**57/317**	**100.0**	**3.71(1.75-7.79)**

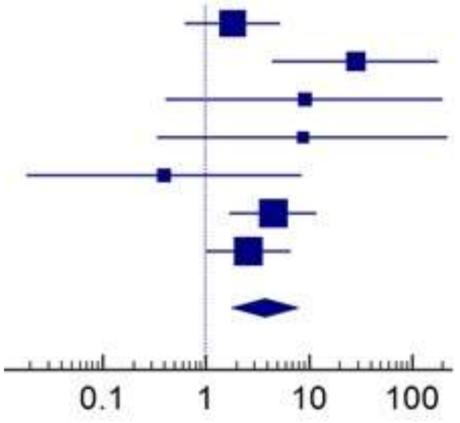

Heterogeneity: df=6, (p=0.14), I^2=37.00%
Test for overall effect Z=3.4, p=0.00

Supplemental Fig 11. Forest plot for the overall outcome in accordance to indication biopsy. The odds ratio (OR) of long-term outcome for the de novo DSA groups. The blue boxes represent the weight of the study, and lines represent the 95% CI for individual studies. The blue diamond at the end represents the pooled OR.

	DSA⁺, outcome⁺/Total DSA⁺	DSA⁻, outcome⁺/Total DSA⁻	Weight %	Odds ratio(95% CI)
Jucaud et al. 2019	9/13	2/27	12.9	28.13(4.37-180.77)
Kovandova et al. 2018	1/8	7/115	11.1	2.20(0.24-20.51)
Kubal et al. 2018	4/27	8/53	16.0	0.98(0.27-3.59)
Tokodai et al. 2018	7/14	0/4	7.8	9.00(0.41-198.22)
Ueno et al. 2016	7/12	2/11	12.7	6.30(0.93-42.73)
San Segundo et al. 2016	0/5	4/23	7.9	0.39(0.02-8.49)
Grabhorn et al. 2015	13/21	6/22	16.0	4.33(1.19-15.69)
Miyagawa-Hayashino et al. 2012	28/32	6/35	15.6	33.80(8.62-132.93)
**Total (95%CI)**	**69/132**	**35/290**	**100.0**	**5.11(1.70-15.33)**

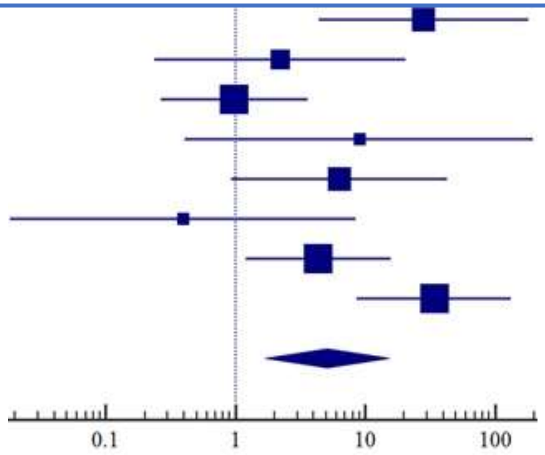

Heterogeneity: df=7, (p=0.00), I^2=57.37%
Test for overall effect Z=2.9, p=0.00

Supplemental Fig 12. Forest plot for the overall outcome in accordance to confounding factors. The odds ratio (OR) of long-term outcome for the de novo DSA groups. The blue boxes represent the weight of the study, and lines represent the 95% CI for individual studies. The blue diamond at the end represents the pooled OR.

	DSA^+, outcome$^+$/Total DSA^+	DSA^-, outcome$^+$/Total DSA^-	Weight %	Odds ratio (95% CI)
Vandevoorde et al.[39] 2018	13/59	56/233	12.6	0.89(0.45-1.77)
Kubal et al.[40] 2018	4/27	8/53	9.5	0.98(0.27-3.59)
Kovandova et al.[44] 2018	1/8	7/115	5.8	2.20(0.24-20.51)
den Dulk et al.[42] 2018	1/18	0/50	3.5	8.66(0.33-222.49)
Levitsky et al.[47] 2016	9/11	39/184	8.2	16.73(3.47-80.61)
Ueno et al.[48] 2016	7/12	2/11	6.9	6.30(0.93-42.73)
Grabhorn et al.[49] 2015	13/21	6/22	9.6	4.33(1.19-15.69)
Del Bello et al.[50] 2015	10/21	22/131	11.2	4.51(1.70-11.89)
Kaneku et al.[41] 2013	9/61	66/688	12.3	1.63(0.76-3.46)
Miyagawa-Hayashino et al.[51] 2012	28/32	6/35	9.2	33.80(8.62-132.93)
O"Leary et al.[52] 2011	21/36	13/37	11.3	2.85(1.00-6.65)
**Total (95%CI)**	**116/306**	**225/1559**	**100.0**	**3.53(1.77-7.07)**

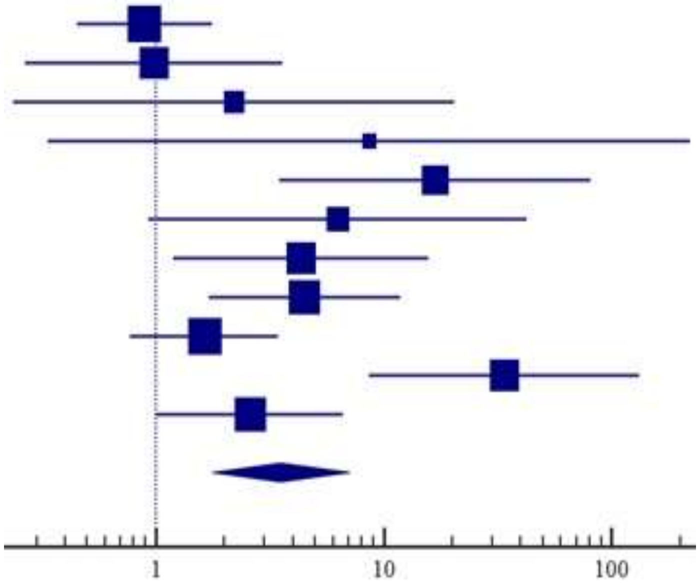

Heterogeneity: df=10, (p=0.00), I^2=46.11%
Test for overall effect Z=3.57, p<0.001

Supplemental Fig 13. Forest plot for the overall outcome in accordance to de novo DSA groups of high methodological quality. The odds ratio (OR) of long-term outcome for the de novo DSA groups. The blue boxes represent the weight of the study, and lines represent the 95% CI for individual studies. The blue diamond at the end represents the pooled OR.

	DSA^{+}, outcome^{+}/Total DSA^{+}	DSA^{-}, outcome^{+}/Total DSA^{-}	Weight %	Odds ratio(95% CI)
Papachristou et al. 2019	10/20	16/45	14.0	1.81(0.62-5.27)
Jucaud et al. 2019	9/13	2/27	9.5	28.13(4.37-180.77)
Tokodai et al. 2018	7/14	0/4	5.2	9.00(0.41-198.22)
Kubal et al. 2018	4/27	8/53	12.6	0.98(0.27-3.59)
den Dulk et al. 2018	1/18	0/50	14.6	8.66(0.33-222.49)
San Segundo et al. 2016	0/5	4/23	4.8	0.39(0.02-8.49)
Ueno et al. 2016	7/12	2/11	5.2	6.30(0.93-42.73)
Grabhorn et al. 2015	13/21	6/22	9.3	4.33(1.19-15.69)
Miyagawa-Hayashino et al. 2012	28/32	6/35	12.6	33.80(8.62-132.93)
O"Leary et al. 2011	21/36	13/37	12.2	2.85(1.00-6.65)
**Total (95%CI)**	**100/198**	**57/307**	**100.0**	**4.44(1.93-10.21)**

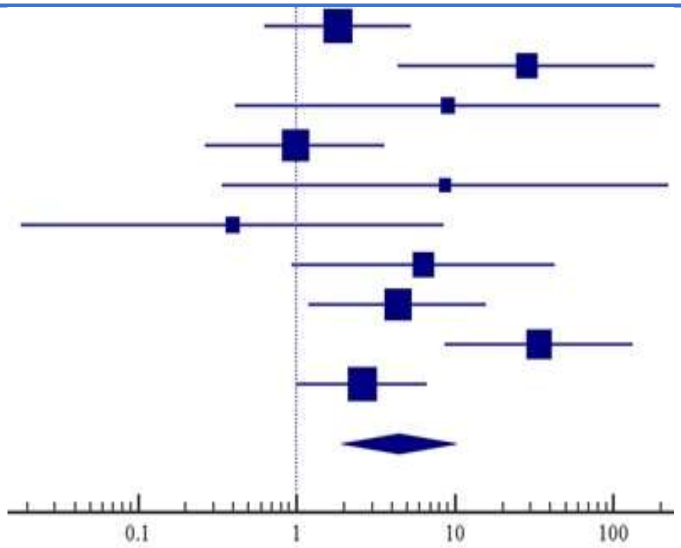

Heterogeneity: df=9, (p=0.00), I^2=52.33%
Test for overall effect Z=3.5, $p<0.001$

Supplemental Fig 14. Forest plot for the overall outcome in accordance to de novo DSA groups of center effect. The odds ratio (OR) of long-term outcome for the de novo DSA groups. The blue boxes represent the weight of the study, and lines represent the 95% CI for individual studies. The blue diamond at the end represents the pooled OR.
